# Factors associated with the evolution of COVID-19 in pregnant women: a Brazilian population-based study^*^

**DOI:** 10.1590/1980-220X-REEUSP-2023-0042en

**Published:** 2023-11-24

**Authors:** Luis Henrique de Andrade, Gustavo Gonçalves dos Santos, Mônica Aparecida de Paula de Sordi, Hélio Rubens de Carvalho Nunes, Cristina Maria Garcia de Lima Parada

**Affiliations:** 1 Universidade Estadual Paulista “Júlio de Mesquita Filho”, Faculdade de Medicina de Botucatu, Programa de Pós-graduação. Botucatu, SP, Brasil.; 2 Universidade de São Paulo, Escola de Enfermagem de Ribeirão Preto, Programa de Pós-graduação em Enfermagem em Saúde Pública. Ribeirão Preto, SP, Brasil.

**Keywords:** COVID-19, Pregnancy Complications, Infectious, Pregnancy, Hospitalization, Maternal Death, Intensive Care Units, COVID-19, Complicaciones Infecciosas del Embarazo, Embarazo, Hospitalización, Muerte Materna, Unidades de Cuidados Intensivos, COVID-19, Complicações Infecciosas na Gravidez, Gravidez, Hospitalização, Morte Materna, Unidades de Terapia Intensiva

## Abstract

**Objective:**

To assess the evolution of COVID-19 among Brazilian pregnant women, identifying sociodemographic and clinical predictors related to admission to ICU - Intensive Care Unit and death.

**Method:**

Cross-sectional, population-based study, carried out with a secondary database, based on data from the Influenza Epidemiological Surveillance Information System. Descriptive analysis was performed, followed by multiple linear regression with Poisson response, adopting critical p < 0.05.

**Results:**

Intensive care admission rates of 28.2% and death rates of 9.5% were identified. Region of residence, gestational trimester, number of comorbidities and respiratory signs and symptoms were associated with the risk of admission to intensive care. Age over 34 years, comorbidities, oxygen saturation equal to or less than 95%, admission to intensive care and ventilatory support, invasive or not, increased the risk of death.

**Conclusion:**

Sociodemographic and clinical predictors showed an association with hospitalization in intensive care and death of pregnant women with COVID-19.

## RESUMO

**Objetivo:**

Avaliar a evolução da COVID-19 entre gestantes brasileiras, identificando-se os preditores sociodemográficos e clínicos relacionados à internação em unidade de terapia intensiva e ao óbito.

**Método:**

Estudo transversal e de base populacional, realizado com banco de dados secundários, a partir de dados do Sistema de Informação da Vigilância Epidemiológica da Gripe. Realizou-se análise descritiva, seguida de regressão linear múltipla com resposta Poisson, adotando-se p crítico <0,05.

**Resultados:**

Identificaram-se taxas de internação em terapia intensiva de 28,2% e de óbito de 9,5%. Região de residência, trimestre gestacional, número de comorbidades e sinais e sintomas respiratórios associaram-se ao risco de internação em terapia intensiva. Idade superior a 34 anos, comorbidades, saturação de oxigênio igual ou inferior a 95%, internação em terapia intensiva e suporte ventilatório, invasivo ou não, aumentaram o risco de óbito.

**Conclusão:**

Preditores sociodemográficos e clínicos mostraram associação com a hospitalização em terapia intensiva e com o óbito de gestantes com COVID-19.

## RESUMEN

**Objetivo:**

Evaluar la evolución de la COVID-19 entre gestantes brasileñas, identificando predictores sociodemográficos y clínicos relacionados con el ingreso en unidad de cuidados intensivos y la muerte.

**Método:**

Estudio transversal, de base poblacional, realizado con una base de datos secundaria, a partir de datos del Sistema de Información de Vigilancia Epidemiológica de Influenza. Se realizó un análisis descriptivo, seguido de regresión lineal múltiple con respuesta de Poisson, adoptando una p crítica <0,05.

**Resultados:**

Se identificaron tasas de ingreso a cuidados intensivos del 28,2% y tasas de mortalidad del 9,5%. La región de residencia, el trimestre gestacional, el número de comorbilidades y los signos y síntomas respiratorios se asociaron con el riesgo de ingreso a cuidados intensivos. La edad mayor de 34 años, las comorbilidades, la saturación de oxígeno igual o inferior al 95%, el ingreso a cuidados intensivos y el soporte ventilatorio, sea invasivo o no, aumentaron el riesgo de muerte.

**Conclusión:**

Los predictores sociodemográficos y clínicos mostraron asociación con la hospitalización en cuidados intensivos y la muerte en gestantes con COVID-19.

## INTRODUCTION

In December 2019, individuals began to develop severe respiratory illness of unknown cause in Wuhan, China. The existence of a beta coronavirus called *Severe Acute Respiratory Syndrome Coronavirus 2* (*SARS-COV-2*) - Severe Acute Respiratory Syndrome 2, popularly known as *Coronavirus Disease 2019* (COVID-19), which causes potentially serious respiratory tract infection and high transmissibility. On January 30 of the same year, due to the growth in the number of cases, a Public Health Emergency of International Concern was declared^(1)^ . In Brazil, the first case of COVID-19 was confirmed on February 26, 2020 and on March 3 of the same year there were already 488 suspected cases notified, two confirmed and 240 discarded in the country^(2)^.

With the evolution of COVID-19 cases, it became important to define priority groups, including pregnant women^(3,4)^, since pregnancy is a period of several physiological changes, and that pregnant women, in situations of infections caused caused by other coronaviruses, such as *SARS-CoV* and *the Middle East Respiratory Syndrome Coronavirus* (*MERS-CoV*) presented frequent complications and the need for admission to the Intensive Care Unit (ICU)^(5,6)^. In most cases of COVID-19, the symptoms presented are mild, with fever and dry cough, however, in some cases, especially in the second half of pregnancy, other symptoms may appear with intensity, such as: fatigue, dyspnea, diarrhea, being It is also possible the occurrence of potentially lethal complications, such as the evolution to Severe Acute Respiratory Syndrome (SARI)^(7)^.

Evidences indicate that pregnant women with severe COVID-19 are at greater risk of evolving to an emergency and/or premature cesarean section and, consequently, to maternal or neonatal death^(8)^. Studies among pregnant women suggest a possible increase in other negative outcomes, such as: spontaneous abortion, premature rupture of membranes, intrauterine growth restriction, fetal distress and premature labor^(9)^. It is noteworthy that the pregnant woman may have her diagnosis of *SARS-COV-2* delayed, as she presents physiological changes typical of pregnancy, masking the condition, such as gestational rhinitis, caused by hyperemia of the nasopharynx, mediated by the high level of estrogen present during pregnancy^(10)^.

In the year 2021, between January and May, 6,416 cases of Severe Acute Respiratory Syndrome (SARS) were reported in pregnant women (257.9 cases per 100,000 pregnant women), of which 4,103 were confirmed as COVID-19 (167.9 cases per 100,000 pregnant women), in 38 cases another diagnosis was confirmed, 1,248 had no definite diagnosis and 1,027 were under investigation. Of the total SARS cases in pregnant women, 505 evolved to death (20.30 deaths per 100,000 pregnant women), 475 of which were confirmed for COVID-19 (19.1 deaths per 100,000 pregnant women)^(3)^.

As for the lethality of COVID-19 in Brazil, in 2020, among hospitalized pregnant women, it was 5.5% and among postpartum women, 12.9%, increasing in the following year to 11.5% and 22.3%, respectively, with the presence of comorbidities increased the risk of worse evolution^(4)^. A systematic review study showed that pregnant women progress more quickly to moderate and severe conditions^(11)^, 1 to 5% need ventilatory support and/or care in the Intensive Care Unit (ICU), with a higher risk of maternal complications being verified mainly in the last two trimesters of pregnancy and in the puerperium^(12)^. As a result, the reasons for maternal death during this pandemic period have increased in developing countries^(13)^, including Brazil, where epidemiological data point to an increase in cases of maternal death from SARS^(14)^.

Considering the gaps in knowledge regarding the evolution of COVID-19 among pregnant women and the possibility of different clinical outcomes, in different places of occurrence, the present study is justified. Thus, the objective was to evaluate the evolution of COVID-19 among Brazilian pregnant women notified in the Influenza Epidemiological Surveillance Information System (SIVEP-Gripe), identifying the sociodemographic and clinical predictors related to hospitalization in the intensive care unit and death.

## METHOD

### Study Design

This is a cross-sectional, population-based study, carried out with a secondary database from the Influenza Epidemiological Surveillance Information System (SIVEP-Gripe).

### Population and Sample

**Figure 1:**
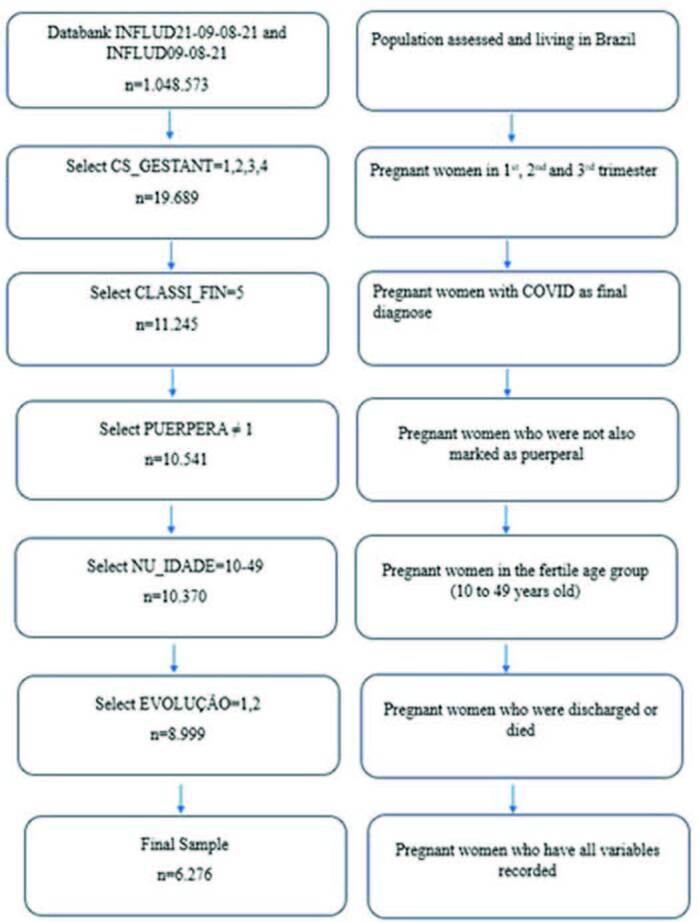
Sample composition, based on data from the Influenza Epidemiological Surveillance Information System. Brazil, 2020–2021

**Source:** Prepared by the authors.

The epidemiological weeks of 2020 were included, between 13 (03/22-28/2020) and 53 (12/27/2020-01/02/2021) and in 2021, between 1 (01/03-09/2021) and 31 (08/01-07/2021), considering data from the entire country. Starting from the complete bank (n = 1,048,573 cases), the selection of pregnant women (n = 19,689 cases) was carried out; then, the final classification of COVID-19 (n = 11,245 cases) was selected. Then, considering that in some situations it was marked as being a pregnant woman and a puerperal woman, possibly due to the evolution of the case, those marked only as pregnant women were maintained (n = 10,541 cases); Then, women in the age group that corresponds to childbearing age, between 10 and 49 years old, were selected, seeking to exclude possible typing errors in the database, since there were some cases of pregnant women with an age incompatible with pregnancy (n = 10,370 cases); finally, cases with annotated final evolution were selected (n = 8,999 cases), since they are related to the outcome of this study (hospitalization in the ICU and death: yes, no). The final sample was defined with the inclusion of participants who had all the information of interest noted down (n = 6,276) (Figure 1).

### Data Collection

Data collection was carried out from July to September 2021, based on SIVEP Gripe data, INFLUD21-09-08-21 and INFLUD09-08-21 databases, made available by the Ministry of Health (MS) of Brazil (Available at: https://opendatasus.saude.gov.br/dataset/bd-srag-2020).

The outcome variables are: ICU admission and death from COVID-19 (dichotomous variables, classified as yes or no). Exposure variables include sociodemographic characteristics: age (years), skin color (white or yellow, brown, black, indigenous); region of residence (southeast, northeast, south, north, midwest) gestational trimester; comorbidities reported at the time of notification (yes, no): heart disease, hematological disease, Down syndrome, liver disease, asthma, diabetes, neurological disease, lung disease, immunosuppression, nephropathy, obesity and number of comorbidities; pregnancy-related diseases (yes, no): gestational diabetes and hypertensive syndromes; signs and symptoms reported at the time of notification (yes, no): fever, cough, odynophagia, dyspnea, respiratory distress, oxygen saturation below 95%, diarrhea, vomiting, loss of smell and taste, and other signs and symptoms (yes, no): runny nose, myalgia, headache, nausea, nasal congestion, lack of appetite, chest pain, chills, weakness, tachycardia, abdominal pain, fatigue, sinusitis, cyanosis of the extremities, lumbar or lower abdominal pain, vaginal bleeding and otalgia; number of signs and symptoms and ventilatory support (no, yes and non-invasive, yes and invasive). Considering that in SIVEP-Gripe the presence of the event is requested, either comorbidity, signs and symptoms, ignored cases (blank) were included in the no group. It is noteworthy that due to the small number of cases and similarity in terms of social aspects, informed pregnant women with yellow skin color were analyzed together with white women.

### Data Analysis and Processing

Initially, a descriptive analysis of the variables related to sociodemography, comorbidities, pregnancy-related diseases and signs and symptoms reported at the time of hospitalization was carried out. Then, multiple linear regression models with *Poisson* response were adjusted through the class of generalized linear models to explain ICU admission and death from COVID-19, including, in the deterministic component of the models, the variables which, separately, associated up at p < 0.10 level with the outcomes. The effect measure adopted was the relative risk, with the respective 95% confidence intervals. In the final multiple regression models, associations were considered statistically significant if p < 0.05. Analyzes were performed using the *Statistical Package for the Social Sciences* (SPSS) 21 software.

### Ethical Aspects

The preservation of ethical aspects was ensured, in accordance with Resolution of the National Health Council N° 510, of April 7, 2016, sole paragraph, which states that they will not be registered or evaluated by the Research Ethics Committee/National Commission system. of Ethics in Research (CEP/CONEP), item II: research that uses publicly accessible information, pursuant to Law N° 12,527, of November 18, 2011. It is noteworthy that the database used is publicly accessible, it does not contain the names of the participants or any other possibility of individual identification of the women, in order to guarantee anonymity. As this is a research with a publicly accessible database, it was not necessary to refer it to the Research Ethics Committee^(15)^.

## RESULTS

The characteristics of the pregnant women included in the study are shown in Table 1.

**Table 1:** Sociodemographic and clinical characteristics of pregnant women participating in the study (n = 6,276) – Brazil, 2020–2021.

Characteristics	No	%
**Age (years)**		
≤ 19	440	7.0
20–34	4148	66.1
>34	1688	26.9
**Skin color**		
Brown	3143	50.1
White or yellow	2722	43.3
Black	362	5.8
Indigenous	49	0.8
**Region of residence**		
Southeast	2464	39.3
North East	1313	20.9
South	1053	16.8
North	766	12.2
Midwest	680	10.8
**Gestational trimester**		
First	564	9.0
Second	1765	28.1
Third	3947	62.9
**Number of comorbidities**		
0	4903	78.1
1	1027	16.4
2	266	4.2
≥ 3	80	1.3
**Number of signs and symptoms**		
0–1	1451	23.1
2–4	3805	60.6
≥ 5	1020	16.3
**Respiratory symptoms**		
Dyspnea		
No	2593	41.3
Yes	3683	58.7
Respiratory distress		
No	3348	53.3
Yes	2928	46.7
Oxygen saturation ≤ 95%		
No	3887	61.9
Yes	2389	38.1
Ventilatory support		
No	3068	48.9
Yes, non-invasive	2418	38.5
Yes, invasive	790	12.6
**Evolution**	**6276**	**100**
ICU		
No	4507	71.8
Yes	1769	28.2
Death		
No	5679	90.5
Yes	597	9.5

**Source:** Prepared by the authors.

Most pregnant women were between 20 and 34 years old; brown skin color; resided in the southeast or northeast regions; was in the third gestational trimester; had no comorbidity; referred between two and four signs and symptoms, including dyspnea, but did not present respiratory distress or oxygen saturation equal to or less than 95% and needed some type of ventilatory support. The proportion of ICU admission and death was 28.2% and 9.5%, respectively. Table 2 is related to the comorbidities and the signs and symptoms presented by the pregnant women. The most frequently reported comorbidities were diabetes, obesity, heart disease and asthma. Among the main signs and symptoms were cough, fever, odynophagia and fatigue (Table 2).

**Table 2:** Prevalence of comorbidities and signs and symptoms of pregnant women participating in the study (n = 6,276) – Brazil, 2020–2021.

Prevalences	No	%
Comorbidities	**1790**	**28.5**
Diabetes	480	7.6
Obesity	404	6.4
Heart disease	356	5.7
Asthma	226	3.6
Immunosuppression	60	1.0
Hypertensive syndrome	57	0.9
Neurological disease	38	0.6
Lung disease	35	0.6
Kidney disease	34	0.5
Hematological disease	27	0.4
Hypothyroidism	20	0.3
Anemia	14	0.2
Liver disease	13	0.2
Syphilis	8	0.1
Down’s syndrome	5	0.1
Cancer	5	0.1
Arthritis	4	0.1
Thrombosis	4	0.1
Signs and symptoms	**16625**	**264.8**
Cough	4466	71.2
Fever	3527	56.2
Odynophagia	1436	22.9
Fatigue	1346	21.4
Loss of smell	1058	16.9
Loss of taste	875	13.9
Myalgia	868	13.8
Nausea or vomiting	794	12.7
Diarrhea	694	11.1
Runny nose	458	7.3
Abdominal pain	443	7.1
Asthenia	140	2.2
Chest pain	110	1.8
Nasal congestion	102	1.6
Backache	66	1.1
Chills	54	0.9
Lack of appetite	53	0.8
Flu like symptoms	50	0.8
Tachycardia	22	0.4
Retro orbital pain	18	0.3
Pain in lower abdomen	17	0.3
Vaginal bleeding	14	0.2
Otalgia	5	0.1
Dysuria	5	0.1
Sweating	4	0.1

**Source:** Prepared by the authors.

Table 3 is related to the bivariate analyzes and multiple regression between the variables of interest and the outcome ICU admission. Variables that were associated with the outcome at p<0.10 were included in the final logistic regression model. In the final model, being ≤ 19 years old, compared to being between 20–34 years old, independently protected pregnant women from ICU admission (RR = 0.78, 95%CI = 0.62–0.99, p = 0.044). Compared to the North region, residing in the Southeast (RR = 1.25, CI95% = 1.04–1.49, p = 0.016) or Northeast (RR = 1.22, CI95% = 1.01–1.47, p = 0.042), being in the third (RR = 1.41, 95%CI = 1.16–1.70, p < 0.001) or second trimester of pregnancy (RR = 1.40, 95%CI = 1.15–1.71, p = 0.001), the number of comorbidities (RR = 1.14, 95%CI = 1.07–1.22, p < 0.001) and symptoms: dyspnea (RR = 1.79, 95%CI = 1.56–2.04, p < 0.001), respiratory distress (RR = 1.14, 95%CI = 1.03–1.28, p < 0.015) and oxygen saturation equal to or less than 95% (RR = 2.12, 95%CI = 1.89–2.38, p < 0.001) were also independently associated with ICU admission. In terms of effect magnitude, the greatest were related to having dyspnea or oxygen saturation equal to or less than 95%, which increased the risk of ICU admission by approximately two times.

**Table 3:** Simple *Poisson* bivariate analysis and multiple regression between variables of interest and the outcome admission to Intensive Care Unit – Brazil, 2020–2021.

Variable	Bivariate analysis			Multiple regression		
	RR	CI95%	P	RR	CI95%	P
**Age years)**						
> 34	1.23	1.11–1.36	**<0.001**	1.09	0.98–1.21	0.100
≤ 19	0.61	0.48–0.77	**<0.001**	0.78	0.62–0.99	**0.044**
20–34	1					
**Skin color**						
Indigenous	0.39	0.17–0.87	**0.021**	0.73	0.32–1.65	0.452
Black	0.89	0.72–1.09	0.255			
Brown	0.82	0.74–0.90	**<0.001**	0.94	0.84–1.06	0.324
White/yellow	1					
**Region**						
Southeast	1.49	1.26–1.75	**<0.001**	1.25	1.04–1.49	**0.016**
South	1.23	1.02–1.48	**0.034**	1.02	0.83–1.27	0.821
Midwest	1.21	0.98–1.48	**0.079**	1.04	0.84–1.29	0.701
North East	1.02	0.84–1.23	0.855	1.22	1.01–1.47	**0.042**
North	1					
**Gestational trimester**						
Third	1.30	1.08–1.58	**0.007**	1.41	1.16–1.70	**<0.001**
Second	1.60	1.31–1.95	**<0.001**	1.09	1.15–1.71	**0.001**
First	1					
**Number of comorbidities**	1.30	1.22–1.38	**<0.001**	1.14	1.07–1.22	**<0.001**
Respiratory symptoms						
Dyspnea	2.79	2.49–3.14	**<0.001**	1.79	1.56–2.04	**<0.001**
Respiratory distress	1.95	1.77–2.15	**<0.001**	1.14	1.03–1.28	**0.015**
Oxygen saturation < 95%	2.97	2.69–3.27	**<0.001**	2.12	1.89–2.38	**<0.001**
**Number of signs/symptoms**	1.01	0.98–1.04	0.422			

**Source:** Prepared by the authors.

Table 4 refers to the death outcome. The variables which were associated with death at p < 0.10 were included in the final logistic regression model. In the final model, independently, residing in the southeast (RR = 0.72, CI95% = 0.55–0.94, p = 0.016) or south (0.60, CI95% = 0.42–0, 84, p = 0.003), in relation to the north region, protected against the occurrence of death, just as there was protection with the increase in the number of signs and symptoms (RR = 0.94, 95%CI = 0.89–0.99, p = 0.010). Also independently, increased the risk of death: being over 34 years old, compared to the age group between 20–34 years old (RR = 1.21, 95%CI = 1.02–1.44, p = 0.027); an increase in the number of comorbidities (RR = 1.17, 95%CI = 1.06–1.30, p < 0.003) or oxygen saturation equal to or less than 95% (RR = 1.24, 95%CI = 1.01–1.53, p = 0.038), having been admitted to the ICU (RR = 2.49, 95%CI = 1.93–3.21, p < 0.001) and needing invasive ventilation (RR = 10.25, 95%CI = 7.11–14.76, p < 0.001) or non-invasive (RR = 2.44, 95%CI = 1.73–3.43, p < 0.001). In terms of effect magnitude, the largest was related to the need for invasive ventilation, which increased the risk of death by approximately 10 times (Table 4).

**Table 4:** Simple Poisson bivariate analysis and multiple regression between variables of interest and death outcome – Brazil, 2020–2021.

Variable	Bivariate analysis			Logistic regression		
	RR	CI95%	P	RR	CI95%	P
**Age years)**						
> 34	1.49	1.25–1.76	**<0.001**	1.21	1.02–1.44	**0.027**
≤ 19	0.55	0.36–0.86	**0.008**	0.89	0.57–1.39	0.605
20–34	1					
**Color**						
Indigenous	0.94	0.35–2.53	0.905	1.25	0.45–3.48	0.671
Black	1.27	0.91–1.78	0.156	1.17	0.83–1.65	0.360
Brown	1.16	0.98–1.38	**0.079**	1.14	0.93–1.39	0.213
White/yellow	1					
**Region**						
Southeast	0.84	0.66–1.06	0.148	0.72	0.55–0.94	**0.016**
South	0.65	0.48–0.87	**0.004**	0.60	0.42–0.84	**0.003**
Midwest	0.76	0.55–1.05	**0.098**	0.80	0.58–1.11	0.179
North East	0.67	0.51–0.89	**0.005**	0.84	0.63–1.11	0.219
North	1					
**gestational trimester**						
Third	0.83	0.62–1.09	0.177			
Second	1.12	0.84–1.50	0.432			
First	1					
**Number of comorbidities**	1.48	1.34–1.63	**<0.001**	1.17	1.06–1.30	**0.003**
Respiratory symptoms						
Dyspnea	3.05	2.48–3.74	**<0.001**	1.08	0.86–1.35	0.531
Respiratory distress	2.49	2.09–2.96	**<0.001**	1.15	0.94–1.39	0.169
Oxygen saturation ≤95%	3.77	3.16–4.49	**<0.001**	1.24	1.01–1.53	**0.038**
**Number of signs and symptoms**	0.96	0.92–1.01	**0.097**	0.94	0.89–0.99	**0.010**
**Intensive care admission**	9.43	7.75–11.47	**<0.001**	2.49	1.93–3.21	**<0.001**
**Ventilatory support**						
Yes, invasive	27.54	20.72–36.62	**<0.001**	10.25	7.11–14.8	**<0.001**
Yes, non-invasive	3.76	2.76–5.12	**<0.001**	2.44	1.73–3.43	**<0.001**
No	1					

**Source:** Prepared by the authors.

## DISCUSSION

The present study made it possible to evaluate the evolution of COVID-19 among Brazilian pregnant women notified in the SIVEP-Gripe, identifying sociodemographic and clinical predictors related to hospitalization in the intensive care unit and death . It identified ICU admission rates of 28.2% and death rates of 9.5%. Among the predictors for ICU admission due to COVID-19, residing in the southeast or northeast regions, when compared to residing in the northern region; being in the second or third trimester of pregnancy, compared to the first trimester; the number of comorbidities and presenting respiratory signs and symptoms: dyspnea, respiratory distress or oxygen saturation equal to or less than 95%, increased the risk of the pregnant woman needing the ICU, while being up to 19 years old was an independent protective factor for this denouement. Being over 34 years old, compared to the age group between 20–34 years old; the number of comorbidities; present oxygen saturation equal to or less than 95%; requiring ICU admission and invasive ventilation did not independently increase the risk of death among pregnant women with COVID-19. Also independently, when compared to residing in the northern region, residing in the southeast or southern regions of the country was a protective factor in relation to death, as well as the number of reported signs and symptoms.

The number of comorbidities is a risk factor identified both when considering the need for hospitalization in the ICU and the evolution to death among pregnant women with COVID-19, the most frequently found in the present study being diabetes and obesity. Clinical comorbidities were also identified in a prospective cohort study conducted in Turkey: they were present in 10 cases (34.5%), with obesity being the main condition (50%), followed by hypothyroidism (40%), so that the authors concluded that individuals with comorbidities are more susceptible to COVID-19, although they indicate the need for further studies on the subject^(16)^. In a study carried out in England, Northern Ireland and Scotland, among pregnant women hospitalized for *SARS-CoV-2,* one third had pre-existing comorbidities, the main ones being obesity, hypertension and diabetes^(17)^. Other studies reinforce that pregnant women with diseases such as hypertension, diabetes mellitus and asthma are more susceptible to the virus and have a more severe course of the disease, leading to respiratory failure and the need for mechanical ventilation^(16-19)^. In contrast, a multinational retrospective cohort study, which included pregnant women with *SARS-CoV-2 infection* from 22 different countries, performed a regression analysis of potential predictors of adverse outcomes and did not identify an association with the presence of chronic comorbidities or obesity^(18)^. Thus, other studies still deserve to address the role of chronic comorbidities in the evolution of COVID-19.

The presence of respiratory signs and symptoms was a predictor of severe disease, which is in line with the scientific literature^(16,18,20,21)^; however, it is noteworthy that both the signs and symptoms associated with COVID-19 described, as to its severity. In Turkey, cough and myalgia were the main initial symptoms reported by pregnant women, while increased temperature, tachypnoea and tachycardia were the most commonly reported abnormal vital signs at the time of hospital admission: 27.6%, 24.1% and 27.6%, respectively^(16)^. In a multinational cohort study, the most common symptom was cough (52.1%), followed by fever (44.1%) and shortness of breath (15.5%), while 24.2% were asymptomatic^(18)^. In the present study, most pregnant women reported between two and four signs and symptoms, and two of the most frequent ones are similar to those mentioned above: fever and cough, while the other two, fatigue and odynophagia, do not.

It is noteworthy, however, that the increase in the number of signs and symptoms was a protective factor in relation to death. Thus, considering the numerous variants of the virus that have already been described and that are yet to come, new research should address the readiness of the care received and not just the cumulative number of signs and symptoms, as a deeper analysis of clinical aspects may bring information that allows understanding better the occurrence and evolution of the disease.

Living in the southeast or northeast regions, when compared to the north, was a risk factor for ICU admission. Another Brazilian study, whose objective was to identify risk factors for adverse outcomes in pregnant and postpartum women with COVID-19, analysing access to health and social risk factors, pointed out that the distribution of ICU beds is not homogeneous in the country, the which may explain why not having a local ICU reduced the risk of hospitalization in these Units^(20)^. Similarly, less ICU admissions in the north region may be due to the lower availability of beds, a plausible explanation when considering another result obtained: residing in the south or southeast regions was a protective factor for death, when compared to the north region.

Other risk factors need to be highlighted: being in the second or third trimester of pregnancy increased the risk of admission to the ICU, maintaining the pattern observed for other respiratory viruses, since in general women at the end of pregnancy are more severely affected^(17,19)^. Age up to 19 years old was a protective factor for ICU admission and age over 34 years old was a risk factor for death, when compared to those between 20–34 years old, probably due to the higher prevalence of comorbidities, such as hypertension and diabetes. The association between severity and comorbidity has been described since the beginning of the pandemic: a multinational retrospective cohort study carried out in 72 Perinatal Centers in 22 countries in Europe, the United States of America, Asia, South America and Australia, between February and April 2020, has already specifically described three cases of death, one of which occurred in a diabetic woman and the other in a hypertensive woman who developed severe pre-eclampsia^(18)^.

In a population-based study carried out in the United Kingdom, 70% of pregnant women were overweight or obese, 40% were aged 35 years or older and one third had pre-existing comorbidities (17). A study carried out in Brazil pointed out: age over 35 years, obesity, diabetes, black skin colour and living in a peri-urban area were factors associated with an increased risk of adverse outcomes, such as spontaneous abortion, premature birth, intrauterine growth restriction, admission in ICU and maternal death. Furthermore, it is noteworthy that the ICU admission and death rates were high: 28.2% and 9.5%, respectively, above that found in international studies, where ICU admission rates were close to 10%^(17,18)^ and maternal death in 0.8%^(18)^. These data show the seriousness of the Brazilian situation and indicate that the unprecedented global humanitarian and health crisis imposed by the pandemic took on an even more dramatic face in the country, adding to the political crisis experienced the adoption of a posture of denial of science and withholding data, disregarding the mourning and suffering of thousands of people^(22)^, culminating in these alarming results.

Finally, it is pointed out that the use of a secondary database constitutes a limitation of this study, since there is dependence on notifying health professionals for their feeding, without researchers being able to control their quality. In addition, as the bank is continuously replenished, there may not have been time to complete the notification, with a note about the need for ICU admission or even the evolution to death, resulting in some underestimation. On the other hand, it is worth highlighting the fact that a population-based database was used, from a country like Brazil, which has continental dimensions.

## CONCLUSIONS

High rates of ICU admission and death were found. Among the predictors for ICU admission due to COVID-19 there were: residing in the southeast or northeast regions, being in the second or third trimester of pregnancy, the number of comorbidities and presenting respiratory signs and symptoms, such as dyspnea, respiratory distress, or saturation oxygen equal to or less than 95%; teenage pregnancy was the only protective factor identified for this outcome. Age greater than 34 years, the number of comorbidities, having oxygen saturation equal to or less than 95%, requiring ICU admission and ventilation, invasive or not, were predictors of death in pregnant women with COVID-19, whereas residing in the South or Southeast regions was a protective factor for this negative outcome.
